# Unveiling the synergistic effect of icariin and azole drugs on *Candida albicans*

**DOI:** 10.3389/froh.2025.1517210

**Published:** 2025-02-13

**Authors:** Barbod Aliaghazadeh, Marina Pascual-Ortiz, Alba Martínez, Veronica Veses, Chirag C. Sheth

**Affiliations:** ^1^ Department of Biomedical Sciences, Faculty of Health Sciences, Universidad Cardenal Herrera-CEU, CEU Universities, Valencia, Spain; ^2^ Department of Pharmacy, Faculty of Health Sciences, Universidad Cardenal Herrera-CEU, CEU Universities, Valencia, Spain; ^3^ Department of Medicine and Surgery, Faculty of Health Sciences, Universidad Cardenal Herrera-CEU, CEU Universities, Valencia, Spain

**Keywords:** antimicrobial resistance, antifungal treatment, flavonoids, *Candida albicans*, icariin

## Abstract

**Introduction and Method:**

The current study explores the synergistic effects of icariin, a flavonoid found in plants of the genus *Epimedium*, in ombination with selected antifungal agents, against *Candida albicans in vitro*.

**Results:**

This flavonoid significantly enhanced the *in vitro* effect of fluconazole, by decreasing the minimum inhibitory concentration against *C. albicans*. This effect was also observed when combining icariin with ketoconazole and itraconazole of the azole family respectively. Interestingly, no activity-enhancing effect was observed when icariin was combined with other classes of antifungals, such as amphotericin B or caspofungin.

**Discussion:**

We conclude that icariin may increase the sensitivity of *C. albicans* to azoles via a cell membrane mediated mechanism, based on our results from FUN-1 microscopy and coincubation with cell wall and cell membrane stressors. Further research is required to explore these effects in clinical isolates, particularly fluconazole-resistant *C. albicans*, with a view towards the clinical application of icariin as a co-adjuvant in antifungal azole therapy.

## Introduction

Growing antimicrobial resistance represents a global challenge, jeopardizing the effectiveness of conventional treatments. The World Health Organization (WHO) has identified antimicrobial resistance as one of the most important challenges in global public health (1). Antimicrobial resistance is predicted to cause as many as 1.9 million deaths in 2050 (up from 1.14 million deaths in 2021) if the trend is not reversed, according to the latest estimations of the Antimicrobial Resistance Collaborators (2). In this context, it is crucial to design and develop protocols suitable for both hospital and community healthcare facilities (3).

Fungal oral infections are remarkably frequent, and many patients are diagnosed by dentists in the clinical setting (3, 4). A number of key pathogenic fungi belong to the genus *Candida*, with *Candida albicans* being the main species responsible for these oral infections (4, 5). *C. albicans* is an opportunistic oral colonizer, causing a diversity of diseases, ranging from oral mucosal superficial infections to life-threating systemic candidiasis (5). The prevalence of oral candidal infections continues to rise as a result of the growing number of chronic patients suffering human immunodeficiency virus infections as well as other vulnerable patient groups in the community, such as those with diabetes mellitus, solid organ transplant, chemotherapy-derived neutropenia and other immunological pathologies (5, 6). *C. albicans* has been included in the WHO fungal priority pathogen as in the critical priority group, thus expressing the urgency in the search of new therapeutic options to combat candida infections (7).

Azoles, polyenes and echinocandins constitute the three main classes of antifungal drugs used against *Candida* infections. Azole antifungal agents (such as fluconazole, itraconazole and ketoconazole) act via the inhibition of the synthesis of ergosterol, a crucial component of fungal cell membrane synthesis, leading to increased membrane permeability and ultimately, cell apoptosis (8). The polyene amphotericin B binds to ergosterol in fungal cell membranes, creating pores that lead to cell lysis. This drug is often reserved for more severe systemic infections due to its significant nephrotoxicity, limiting its widespread usage (9). Caspofungin, from the echinocandin family, inhibits β-(1,3)-D-glucan synthase, weakening the cell wall and causing the cell to lyse. Caspofungin is most typically utilized against infections that have displayed resistance to other treatments, but it can also be associated with serious side effects (10). Therefore, fluconazole (FCZ) remains the preferred treatment for oral candidiasis due to its good oral bioavailability and lower toxicity. Unfortunately, azole resistance is particularly prevalent and extensively documented due to the widespread use of azoles in clinical practice (11). Resistance to polyenes and echinocandins is also increasing alarmingly in many clinical isolates of *C. albicans* and other *Candida sp.* (5).

Faced with the imminent antimicrobial resistance era, where current therapeutic options may become increasingly ineffective, three lines of action can be defined; (i) the discovery of new antimicrobials; (ii) the use of natural compounds alone or as co-adjuvants for existing antimicrobials; and (iii) the development of novel alternative therapies based on bacteriophages, monoclonal antibodies or vaccines (12). The use of combination therapies of antimicrobial agents, with adjuvants of natural origin may be employed to increase effectiveness and reduce the antibiotic doses necessary for treatments, thereby preventing the novel emergence of antimicrobial resistant microorganisms. Natural compounds found in plant extract therapies have been utilized for centuries and show few side effects, with interesting antibacterial and antifungal activities (13, 14). Specifically, the main components described in literature with proven antifungal activities are phenolic compounds, flavonoids, polyphenols, terpenoids and saponins (15).

The current study explores the synergistic effects of icariin, a flavonoid found in plants of the genus *Epimedium,* in combination with selected antifungal agents, against *C. albicans in vitro*. Icariin is known for its varied and wide-ranging pharmacological activities, including neuroprotection and anti-atherosclerosis (16). Our research group has recently characterized a synergistic effect between this flavonoid and β-lactam antibiotics against *Staphylococcus aureus in vitro* (17). To the best of our knowledge, icariin's activity as a potential antifungal agent has not yet been investigated. In this study, we explore the utility of icariin as a potential natural adjuvant in antifungal treatments against *C. albicans in vitro*.

## 2 Materials and methods

### 2.1 *Candida albicans* strain and growth conditions

Yeast strain *C. albicans* used in this study was obtained from the Spanish Type Culture Collection (CECT 11821). The inoculum was stored in yeast extract peptone dextrose (YPD) broth containing 20% glycerol at −20 °C. Pure colonies were obtained from a frozen stock by streaking an inoculum and incubating at 30 °C for 24–48 h on YPD agar plates.

### 2.2 Icariin preparation

Purified icariin was purchased from Merck (catalog number: I1286-100MG) and dissolved in dH_2_O at a concentration of 10 mg/ml. Icariin stock solutions were freshly prepared 24 h before each experiment and aliquots of 100 μl were pipetted into 20 ml YPD agar plates for a final concentration of 50 µg/ml and allowed to dry at room temperature in a sterile laminar flow cabinet as previously described (17).

### 2.3 Antifungal susceptibility testing

Minimum inhibitory concentrations (MICs) for FCZ (0.016–256 μg/ml, Liofilchem catalog number: 92147), caspofungin (0.002–32 μg/ml, Liofilchem catalog number: 92154), and amphotericin B (0.002–32 μg/ml, Liofilchem catalog number: 92153) were determined using test strips according to the manufacturer's instructions. Plates containing icariin were prepared prior to the application of the antifungal strips.

Serial dilution spot assays were carried out using YPD agar plates containing 3 µg/ml FCZ (Thermo Scientific, catalog number 15365008), 0.2 μg/ml caspofungin (Merck, catalog number SML0425-5MG), or 0.5 μg/ml amphotericin B (Cayman Chemical, catalog number 11636), with or without 50 μg/ml icariin. Yeast cultures were incubated overnight in YPD broth at 30°C, diluted to an optical density at 600 nm (OD_600_) of 0.4, and grown to the log phase. Four 10-fold dilutions (10^6^ to 10^3^ cells/ml) were prepared before spotting 5 µl of each one the plates. Growth was evaluated after incubating the plates for 48 h at 30°C.

For additional azole testing the same serial dilution conditions were replicated with plates containing 3 µg/ml FCZ, itraconazole 0.064 µg/ml (Sigma Aldrich, catalog number PHR1834), and ketoconazole 0.032 (Sigma Aldrich, catalog number PHR1385) µg/ml in the presence or absence of icariin 50 µg/ml.

*In vitro* interaction between FCZ and Icariin was performed following the checkerboard method (18). Briefly, 50 µl of icariin dissolved in YPD medium at a concentration of 200 µg/ml were added to column 5 (A–F) of a 96-well plate. Two-fold serial dilutions were then prepared, with 100 µg/ml icariin in column 4 (A–F), 50 µg/ml in column 3 (A–F), and 25 µg/ml in column 2 (A–F). Column 1 was left with 50 µl YPD medium only. Next, and overnight yeast culture diluted to an OD_600_ of 0.4, and grown to the log phase was used as diluent to prepare FCZ solutions at 6, 3, 1.5, 0.75, and 0.375 µg/ml. Then, 50 µl of each FCZ dilution was transferred to corresponding wells: 6 µg/ml to row A (columns 1–5), 3 µg/ml to row B (columns 1–5), 1.5 µg/ml to row C (columns 1–5), 0.75 µg/ml to row D (columns 1–5) and 0.375 µg/ml to row E (columns 1–5). Well 1F was used a growth control. Plates were incubated at 30°C and absorbance at 600 nm was monitored after 6 and 24 h.

### 2.4 Viability test of yeast cells

Aliquots of 1 ml of exponentially growing cells cultured under four different conditions: YPD broth, YPD broth with 50 µg/ml icariin, YPD broth with 3 µg/ml FCZ, and YPD broth with 3 µg/ml FCZ plus 50 µg/ml icariin, were stained with 1 µl of 10 µM FUN-1 (purchased from Thermo Fisher Scientific catalog number F7030) (19). Analysis using a Leica DM6 B Fluorescent Microscope was performed after 15 and 30 min of incubation with the dye at 30°C and images were acquired using excitation wavelengths of 573 and the emission wavelength of 591 nm.

Propidium Iodide staining was used as described elsewhere (17) with the following modifications: yeast cultures were incubated with icariin (50 µg/ml) and/or fluconazole (3 µg/ml) at 30 °C for 6 h. Then, 1 ml of each culture was centrifuged at 3,500 rpm for 5 min, resuspended in PBS, and washed twice. After adding 20 µl of propidium iodide (Sigma-Aldrich, 79214), cells were incubated in the dark at room temperature for 15 min. *Candida* cells were then centrifuged again, resuspended in 100 µl PBS and immediately visualized by fluorescent/visible light microscopy. In parallel, yeast cultures incubated with amphotericin B (100 µg/ml) were also stained following the same protocol and used as a positive control for cell death.

### 2.5 Growth curves

*C. albicans* colonies were incubated overnight in YPD broth at 30°C, then diluted to an OD_600_ of 0.4, antifungals were then added at the following concentrations: 3 µg/ml FCZ, 0.2 µg/ml caspofungin, or 0.5 µg/ml of amphotericin B, with or without 50 µg/ml icariin. The OD_600_ was measured at 3, 6, and 9 h using a UV-visible spectrophotometer (Thermo Scientific GENESYS 20).

### 2.6 Cell envelope stressors

To qualitatively assess the effect of icariin on antifungal sensitivity in the presence sodium dodecyl sulfate (SDS; Sigma catalog number 75746-250G) and Calcofluor-white (CFW, Sigma catalog number 18909-100ML-F), serial dilution spot assays were done on YPD agar plates containing 2 µg/ml fluconazole (reduced dosage in response to additive effect of SDS), 50 µg/ml icariin and with/without 0.017% SDS or 75 µg/ml CFW. Yeast cultures were incubated overnight in YPD broth at 30 °C, diluted to an OD_600_ of 0.4, and grown to the log phase. Four 10-fold dilutions (10^6^ to 10^3^ cells/ml) were prepared before spotting 5 µl of each one onto the plates. Images were captured using LI-COR Odyssey® M imaging system after incubating the plates for 48 h at 30°C.

### 2.7 Statistical analysis

All experiments were carried out in triplicate and repeated in at least three independent trials. Paired *t*-test and *p* values were calculated using Microsoft Excel.

## 3 Results

### 3.1 Evaluation of the antifungal effect of the flavonoid icariin on *Candida albicans*

**Figure 1:**
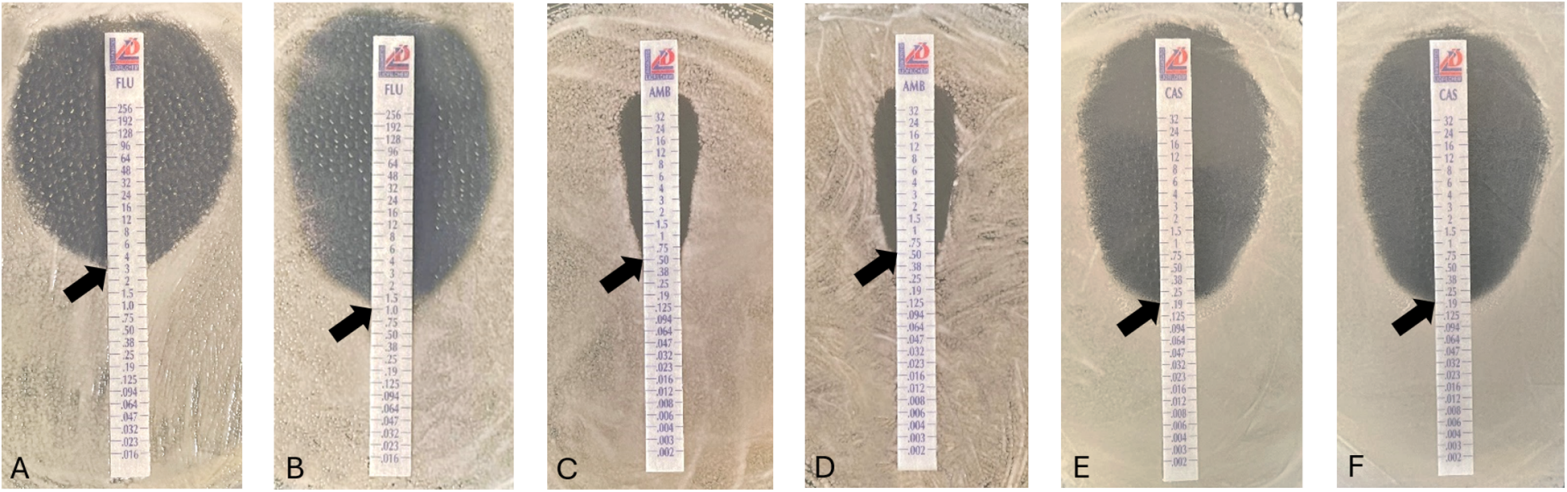
Antifungal susceptibility of *Candida albicans* in the presence of icariin. Minimum Inhibitory Concentration (MIC) evaluated by E-test strips diffusion placed on YPD-agar plates. Effect of FCZ on yeast cells alone **(A)** and in combination with icariin 50 µg/ml **(B)**, amphotericin B **(C)** amphotericin B and 50 µg/ml icariin **(D)** and caspofungin alone **(E)** or in combination with 50 µg/ml icariin **(F)** Observed MIC values indicated with black arrows on all images: 3.0 µg/ml **(A)**, 1 µg/ml **(B)**, 0.5 µg/ml **(C,D)**, 0,19 µg/ml **(E,F)**.

Gradient strip testing is a conventional phenotypic assessment methodology commonly used in clinical practice (20). In this context, gradient diffusion strips were used to assess the potential antifungal activity of the flavonoid icariin, in combination with antifungals from different families (azoles, polyenes and echinocandins), against *C. albicans* (Figure 1).

Activity of caspofungin against *C. albicans* was evaluated in combination with icariin (50 µg/ml), with no differences detected in the minimum inhibitory concentrations (MIC) of the antifungal observed, with a value of 0.19 µg/ml, with or without the flavonoid. Similarly, a lack of effect was also observed with amphotericin B, a polyene, as the MIC value was found to remain unchanged both in the presence and absence of icariin (50 µg/ml) at 0.5 µg/ml. Notably, *C. albicans* showed a significant increase in sensitivity when incubated with fluconazole in combination with icariin (50 µg/ml), as MIC decreased from 3 µg/ml to 1 µg/ml (*p* < 0.00001). The potentiation factor of icariin in combination with fluconazole was calculated to be 3. These data indicate that the flavonoid has a synergistic effect with the azole drug, enhancing its activity threefold (21).

**Figure 2:**
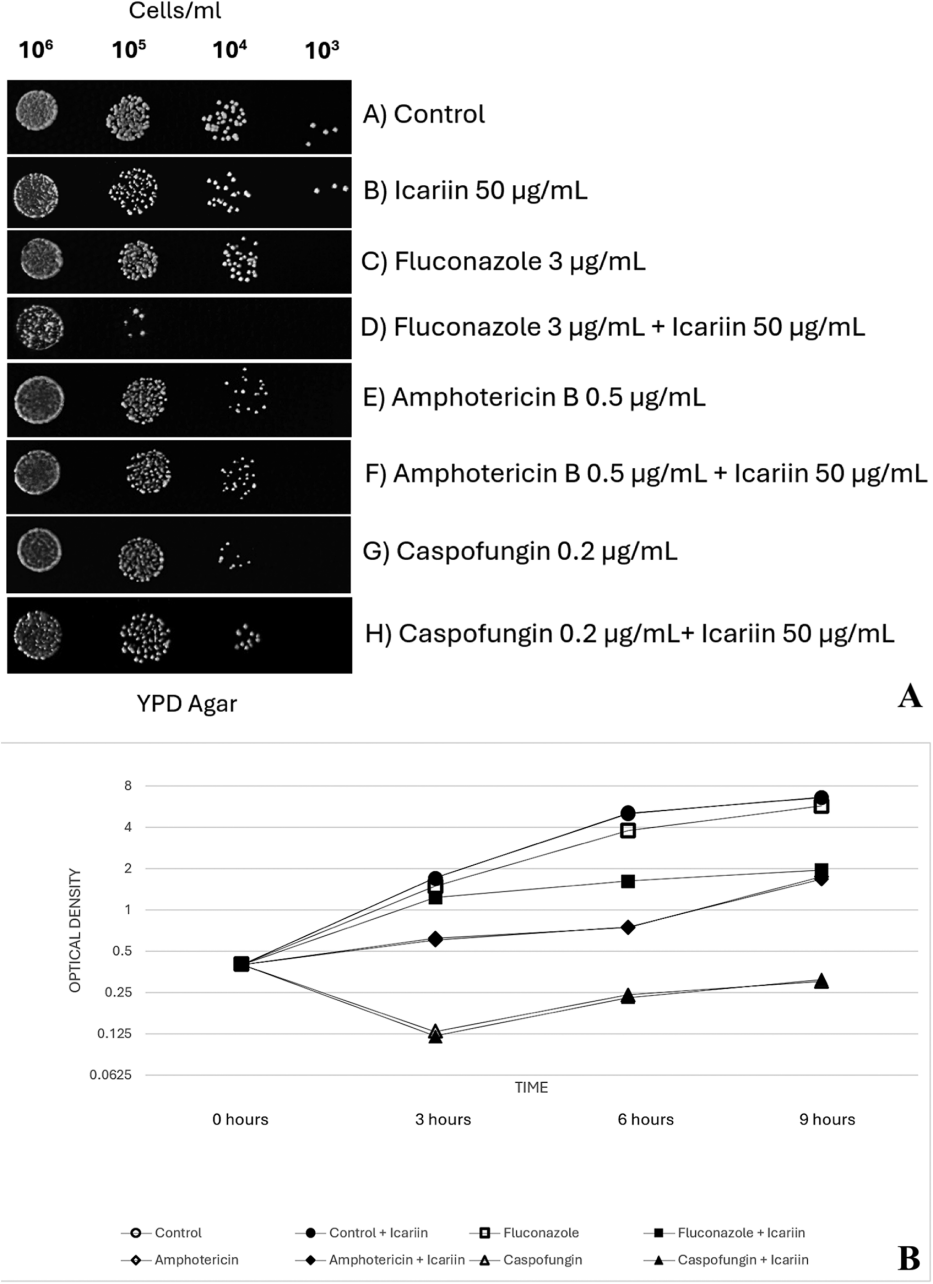
**(A)** Effect of FCZ, amphotericin B and caspofungin on icariin-treated cells. Yeast cells (10^6^ to 10^3^ cells/ml) spotted on YPD-agar plates under different conditions: untreated control **(A)**, 50 µg/ml icariin **(B)**, 3 µg/ml FCZ **(C)**, 3 µg/ml FCZ and 50 µg/ml icariin **(D)**, 0.5 µg/ml amphotericin **(E)**, 0.5 µg/ml amphotericin and 50 µg/ml icariin **(F)**, 0.2 µg/ml caspofungin **(G)**, and 0.2 µg/ml caspofungin with 50 µg/ml icariin **(H)** Plates were photographed after 48 h of incubation at 30 °C. **(B)** Growth of *Candida albicans* in the presence of icariin and antifungals. Optical density measured at 600 nm (OD_600_). Values were obtained at 3, 6, and 9 h of incubating exponentially growing cells at 30 °C in YPD broth. No significant effect was observed when cells were grown in media supplemented solely with icariin (50 µg/ml) in the absence of antifungals (*p* values = 1.00, 0.58, and 0.69 at 3, 6, and 9 h, respectively). Adding icariin (50 µg/ml) to caspofungin (*p* values = 0.58, 0.62, and 0.60) or amphotericin B (*p* = 0.79, 0.92, and 0.68) did not result in statistically significant changes in growth compared to each antifungal alone. However, cells exposed to fluconazole in combination with icariin (50 µg/ml) exhibited significantly reduced growth compared to fluconazole alone (*p* values = 0.034, 0.004, and 0.002 at 3, 6, and 9 h, respectively).

Additional exploration of the effects of icariin on the three primary families of antifungal drugs employed in this study (azoles, polyenes and echinocandins) was carried out via serial dilution trials with *C. albicans* cells at four 10-fold dilutions (10^6^ to 10^3^ cells/ml) in the presence and absence of icariin and a combination of each antifungal (Figure 2A). Coinciding with previous results, a combination of fluconazole (3 µg/ml) and icariin (50 µg/ml) reduced the growth of *C. albicans* cells further than just the presence of the antifungal alone, by dropping the growth of cell colonies from the 10^4^ dilution to the 10^5^ dilution. However, fungal cells exposed to amphotericin B (0.5 µg/ml) and caspofungin (0.2 µg/ml) experienced the same amount of growth until the 10^4^ dilution in both the presence and absence of icariin. Control cells with and without the flavonoid grew to the final dilution of 10^3^.

To quantitatively analyze the effect of icariin when administered along with the antifungals on the growth inhibition of *C. albicans*, OD_600_ was measured following co-incubation of yeast cells with icariin, each antifungal at MIC or a combination of antifungal at MIC and icariin (Figure 2B). Control cells showed a normal growth pattern with an active exponential phase of 6 h before reaching the stationary phase. No effect was observed when the *C. albicans* cells were grown in media supplemented solely with icariin (50 µg/ml) in the absence of antifungals (*p* = 1.00 at 3 h, *p* = 0.58 at 6 h and *p* = 0.69 at 9 h).

When caspofungin was used, OD_600_ decreased at 3 h, and this effect was maintained until stationary phase. On the other hand, exposure of amphotericin B led to an increased OD_600_, but lower than in the control cells, as well as a delayed exponential phase that started at 6 h. In this scenario, adding icariin (50 µg/ml) to caspofungin (*p* = 0.58, *p* = 0.62, *p* = 0.60) or amphotericin B (*p* = 0,79, *p* = 0.92, *p* = 0.68) did not result in statistically significant changes in the growth pattern of *C. albicans* compared to each antifungal alone at 3, 6 or 9 h.

When FCZ was added to the media, *C. albicans* cells showed the same growth pattern as control cells, although OD_600_ was lower at 3, 6 and 9 h. However, a combination of fluconazole plus icariin (50 µg/ml) led to a more remarkable decrease in growth, with an exponential phase lasting only 3 h. OD_600_ values for *C. albicans* cells exposed to FCZ in combination with icariin (50 µg/ml) were statistically lower than those for antifungal alone, at 3, 6 and 9 h (*p* = 0.034, *p* = 0.004, *p* = 0.002, respectively).

**Figure 3:**
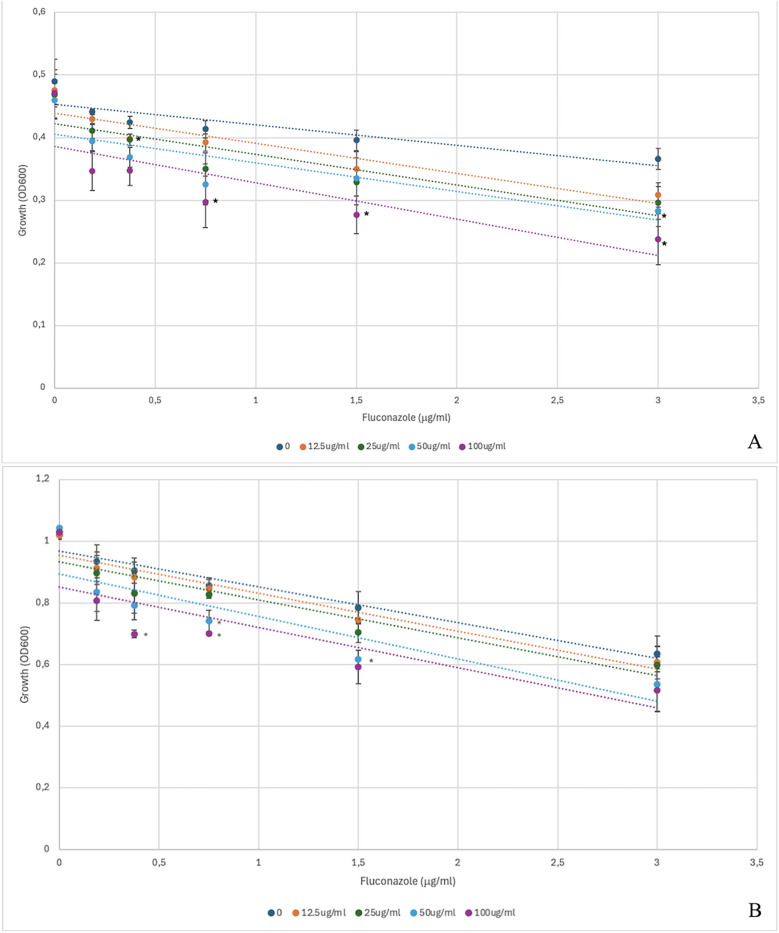
Results of the checkerboard assay at 6 h **(A)** and 24 h **(B)**. *Candida albicans* growth (OD_600_) is plotted against increasing concentrations of fluconazole. The trendlines denote differences in growth for increasing concentrations of icariin. *=*p* > 0.05 as compared to 0 icariin control.

Checkerboard tests were carried out to further characterize the synergistic effect of icariin with fluconazole (Figure 3). We demonstrate that increasing concentrations of icariin enhance the inhibitory effect of FCZ, both at the 6-h (Figure 3A) and 24-h (Figure 3B) timepoints. At the 6-h timepoint, the inhibition was found to be statistically significant for 100 μg/ml at 3 different concentrations of FCZ (3, 1.5 and 0.75 μg/ml), whilst the addition of 50 μg/ml icariin statistically significantly reduced growth in 3 μg/ml FCZ, as compared to the control containing no icariin (Figure 3A). Following 24 h incubation we observe a similar enhancement of FCZ-induced growth inhibition by increasing icariin concentrations. Statistically significant inhibition was observed for the highest icariin concentrations when combined with 0.375 and 0.75 μg/ml FCZ, respectively. It was also observed that 50 μg/ml icariin statistically significantly enhanced the fluconazole activity at 1.5 and 0.75 μg/ml respectively (Figure 3B). When *C. albicans* was incubated with icariin on its own, growth was comparable to the control and there were no statistically significant differences.

### 3.2 Effect of icariin as a synergistic agent alongside other azole antifungal drugs

**Figure 4:**
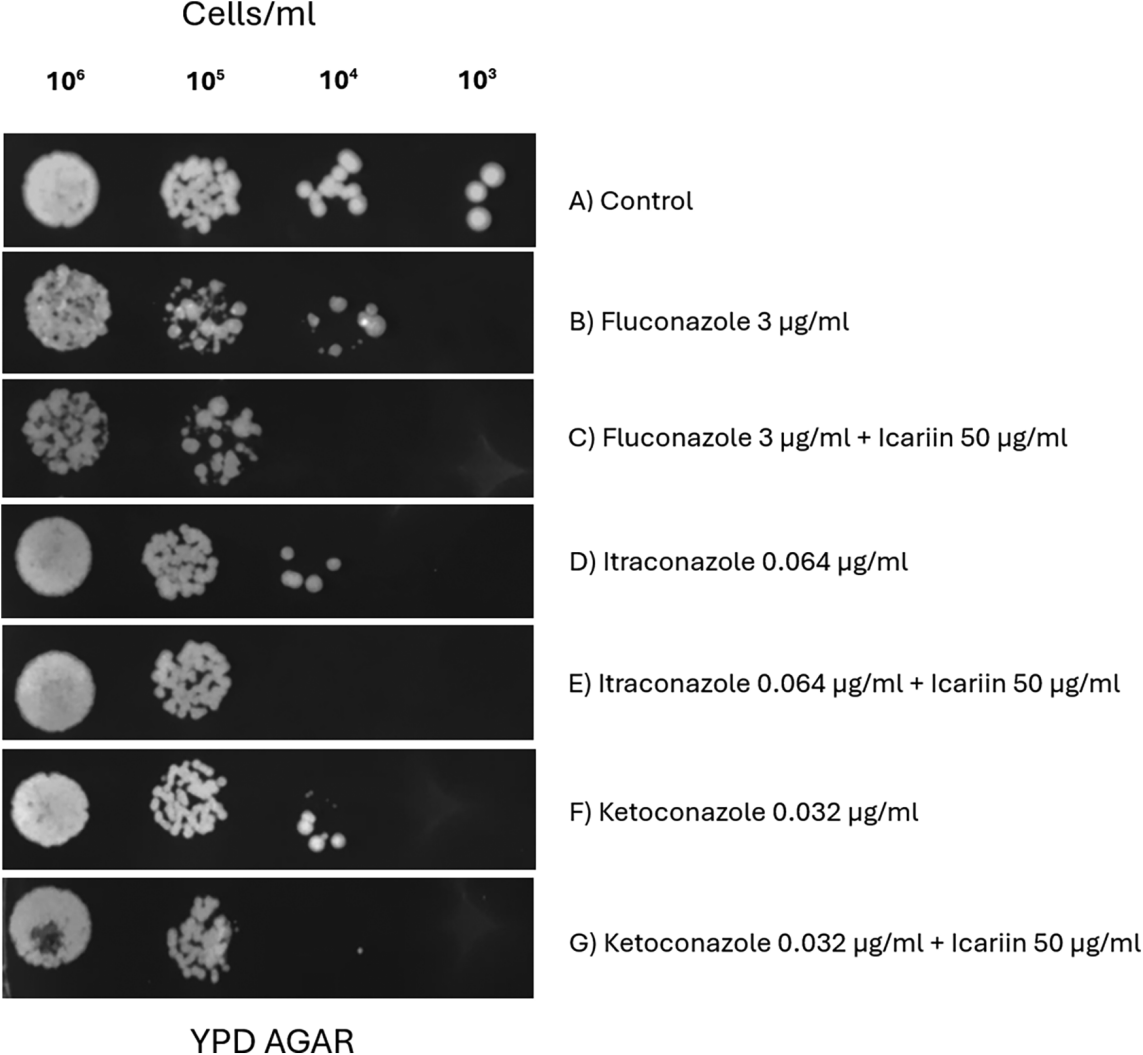
Evaluation of icariin's synergy with different azole antifungals. Serial dilution spot assay of *C. albicans* cells (10⁶ to 10³ cells/ml) grown on YPD agar **(A)**, 3 µg/ml FCZ with and without 50 µg/ml icariin **(C,B)**, 0.064 µg/ml itraconazole only **(D)** or in presence of 50 µg/ml icariin **(E)**, 0.032 µg/ml ketoconazole **(F)** and a combination of 0.032 µg/ml ketoconazole and 50 µg/ml icariin **(G)**.

Since a synergistic effect was detected with FCZ, two additional azole drugs were selected to explore if this effect was also seen with other drugs belonging to the azole family. For that reason, serial dilution trials were carried out at four 10-fold dilutions (10^6^ to 10^3^ cells/ml) with *C. albicans* cells in the presence of fluconazole 3 µg/ml, itraconazole 0.064 µg/ml, and ketoconazole 0.032 µg/ml (Figure 4). We showed that the presence of icariin inhibited the growth of *C. albicans* cells with ketoconazole and itraconazole, similarly to what we observed with FCZ, at 10^4^ and 10^5^ dilutions.

### 3.3 Permeability assay of icariin-treated cells

**Figure 5:**
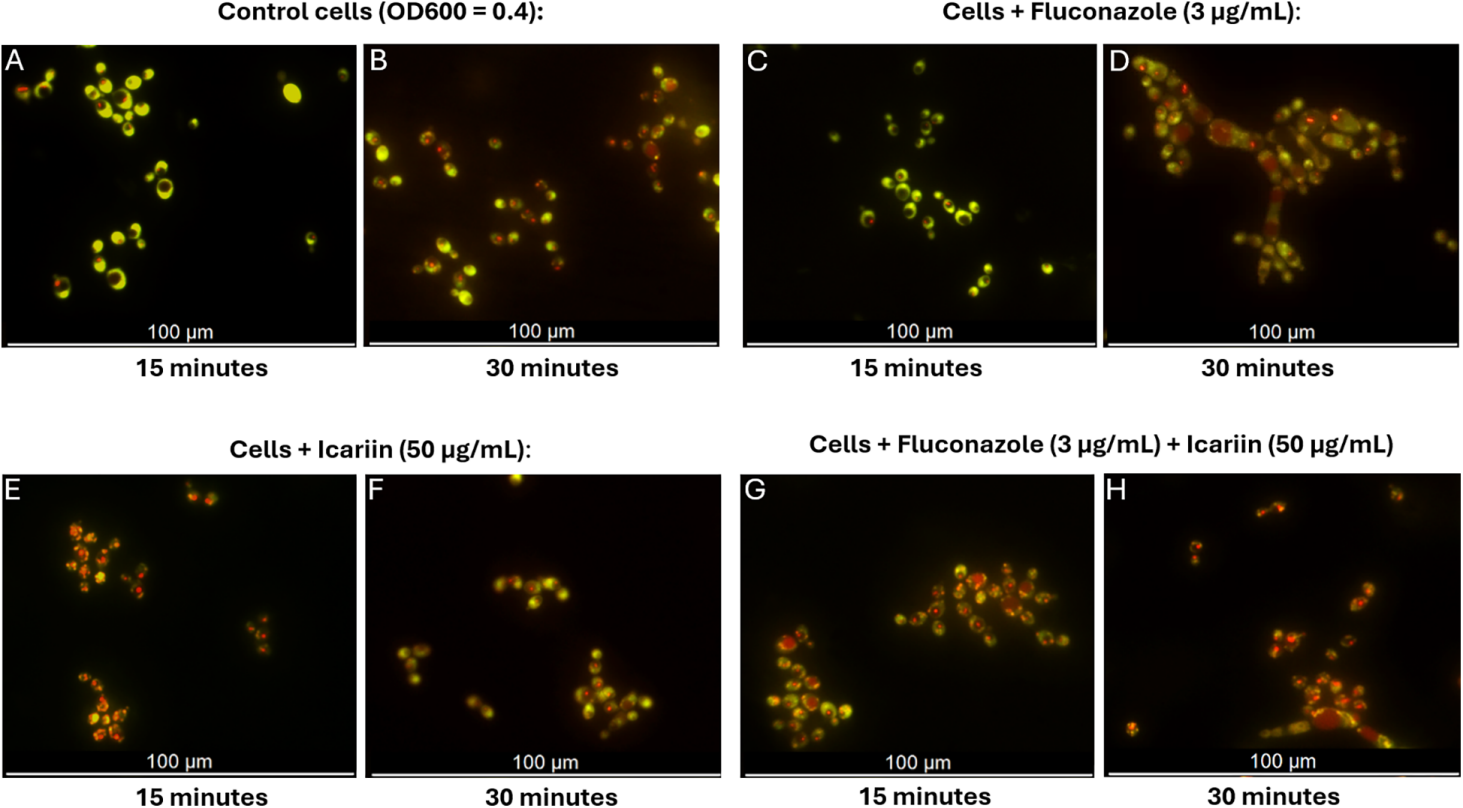
Fluorescent microscopy assessment of permeability of icariin-treated cells. Microscopy images of *C. albicans* cells stained with FUN-1 following cultivation in different conditions: YPD broth **(A,B)**, medium containing 3 µg/ml FCZ **(C,D)**, 50 µg/ml icariin **(E,F)**, or a combination of icariin and FCZ **(G,H)**. Images were captured at 15 and 30 min, scale bar 100 µm.

The effect of icariin on the fungal cell envelope was determined using FUN-1 dye uptake (Figure 5). FUN-1 is a fluorescent dye that serves as an indicator of cell membrane permeability and viability (19). It diffuses into fungal cells, where it is converted to fluorescent red cylindrical intravacuolar structures (CIVS) in metabolically active cells, while dead cells remain green.

In the presence of icariin, orange/red fluorescence appeared earlier (15 min) (Figure 5E), indicating faster dye entry compared to both control cells and fluconazole-treated cells in the absence of icariin which remained predominantly green at that time point (Figures 5A and C). This early red fluorescence detection suggests that icariin may enhance cell permeability, allowing the dye to penetrate the cells more rapidly, regardless of the presence of other antifungal agents. After 30 min samples supplemented with icariin exhibited similar red fluorescence to the FCZ treated cells, thus confirming that icariin has no effect on the metabolic activity of *C. albicans* (Figure 5F).

**Figure 6:**
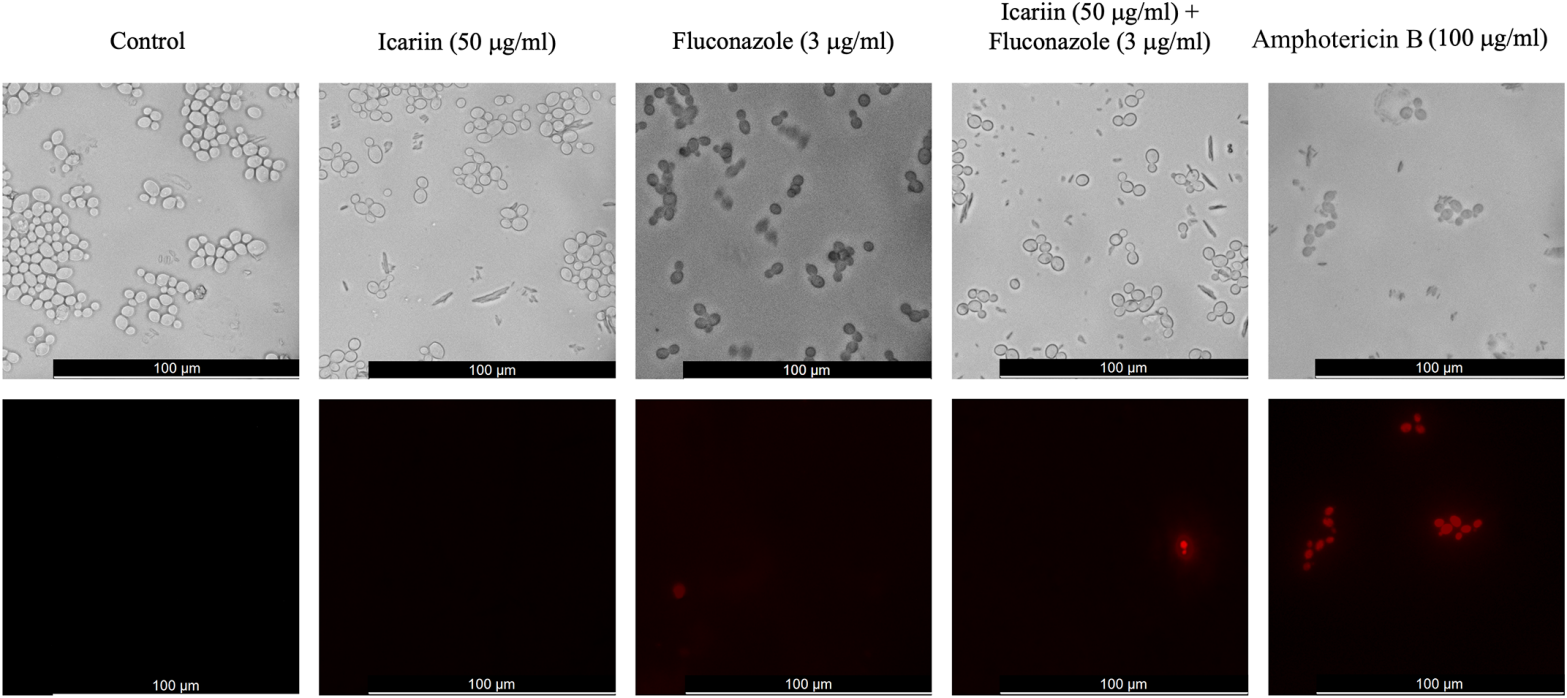
Cell viability assay of icariin-treated cells. Fluorescent microscopy images of *C. albicans* cells stained with propidium iodide following incubation in different conditions. From left to right: YPD broth, medium containing 50 µg/ml icariin, 3 µg/ml FCZ, a combination of icariin and FCZ, or 100 µg/ml amphotericin B. Images were captured after 6 h of incubation. Top panels show phase contrast images and bottom panels red fluorescent images. Scale bar 100 µm.

The viability of cells treated under the current experimental conditions was assessed using propidium iodide staining. Propidium iodide accumulates in dead or dying cells and is visualized as red fluorescence. After 6 h, control cells were observed to be alive. Co-incubation with icariin alone was comparable to the control, whilst 1,3% (1/75) and 1,4% (1/69) of cells treated with FCZ alone and FCZ+ icariin were found to be dead or dying. Amphotericin B was used as a positive control for this experiment (Figure 6).

### 3.4 Analysis of fungal cell wall and cell membrane integrity in presence of stressors

Because azoles disrupt ergosterol synthesis, destabilizing the cell membrane, we next assessed whether icariin affects the same cellular target. We examined the growth of *Candida albicans* cells in response to icariin and SDS, a cell membrane permeabilizer (22).

**Figure 7:**
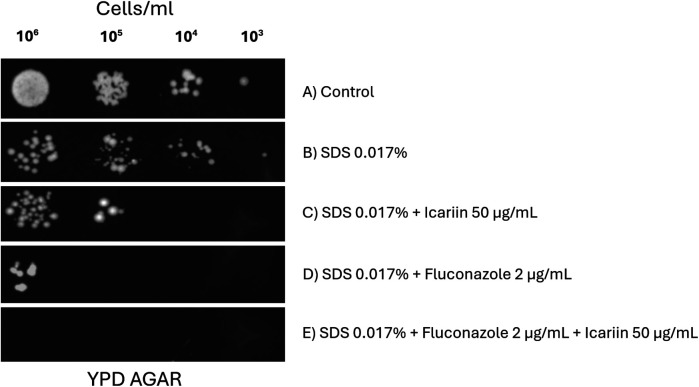
Effect of icariin on cell membrane stress sensitivity. Yeast cells (10^6^ to 10^3^ cells/ml) spotted on YPD-agar plates under different conditions: untreated control **(A)**, 0.017% SDS **(B)**, 0.017% SDS and 50 µg/ml icariin **(C)**, 0.017% SDS and 2 µg/ml FCZ **(D)**, and 0.017% SDS, 2 µg/ml FCZ and 50 µg/ml icariin **(E)** Plates were photographed after 48 h of incubation at 30 °C.

Figure 7 shows yeast cells treated with SDS alone, and in combination with icariin and/or fluconazole. A reduction of growth caused by the detergent is enhanced when icariin is present (Figures 7B,C) suggesting that icariin may target the cell membrane, similarly to SDS. According to this result, the additive effect observed when the antifungal fluconazole and SDS were added to the media (Figure 7D) was increased when icariin was present (Figure 7E) leading to a complete inhibition of growth. This result indicates that the flavonoid targets the fungal cell membrane.

**Figure 8:**
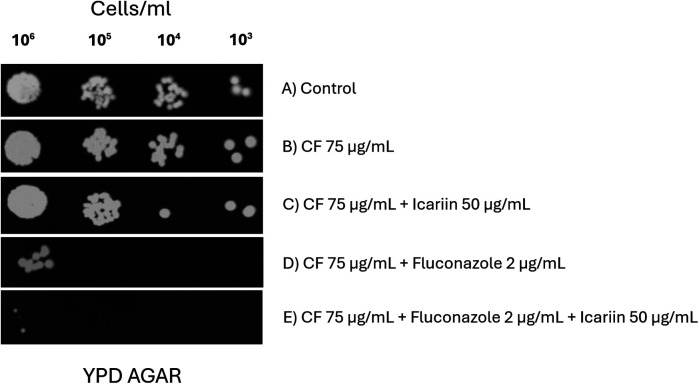
Effect of icariin in combination with the cell wall stressor calcofluor-white. Serial dilution spot assays of *C. albicans* cells grown on YPD media **(A)** supplemented with 75 µg/ml Calcofluor-white (CFW) **(B)**, 75 µg/ml CFW and 50 µg/ml icariin **(C)**, 75 µg/ml CFW and 2 µg/ml FCZ **(D)** 75 µg/L CFW and 2 µg/ml FCZ and 50 µg/ml icariin **(E)** Results were recorded after 48 h of incubation at 30 °C.

Although no synergy between icariin and caspofungin (a glucan synthase inhibitor) was detected (Figure 1), the possibility of icariin targeting cell wall was also investigated. Yeast cells were spotted on agar plates containing Calcofluor-white, a known cell wall stressor (Figure 8) (22). No reduction of growth was observed when cells were exposed to CFW and icariin (Figure 8C) indicating that these agents might use different cellular targets. Consistent with our previous results, the effect of FCZ was enhanced in presence of icariin (Figures 8D,E) regardless of the presence of CFW, confirming the role of icariin as membrane destabilizing agent.

## 4 Discussion

The use of natural compounds as antifungal treatments has been extensively documented in the literature (13–15, 23). Garlic, green tea, propolis, curcumin, licorice root, cinnamon, resveratrol, ginger, and berberine have proven useful in the treatment of *C. albicans* in the context of oral candidiasis (24). Low risk of side effects and wide accessibility of natural compounds represent their main advantages. On the other hand, lack of standardization in the formulations and limited data from randomized clinical trials in humans are preventing widespread use in clinical settings, including dental clinics. In this context, in recent years a lot of effort has been devoted to identifying active ingredients within herbal extracts as well as quantifying the MIC of each compound (15, 25). *In vitro* studies on flavonoids have shown consistently satisfactory results, not only alone, but also used as adjuvants in synergy with current antifungal drugs (26, 27). Amongst flavonoids subclasses, flavonols (quercitin, canthin-6-one) and chalcones (lichochalcone-A) have shown adequate antifungal activities *in vivo* (28).

Previous studies in our lab demonstrated a synergistic effect of icariin with beta-lactamic antibiotics against laboratory strains of *Staphylococcus aureus* as well as methicillin resistant *S. aureus* clinical strains (17). Icariin is a 8-prenyl derivative of kaempferol 3,7-O-diglucoside, a member of the flavonol subfamily (29). In this study, we sought to evaluate the potential antifungal activity of icariin against a *C. albicans* type strain, as an initial step towards development of future strategies to combat the rising incidence of antifungal resistance in oral clinical isolates of *Candida* species. In order to achieve this, we evaluated the antifungal activity of icariin alone, against a *C. albicans* laboratory type strain. Similarly to our previous observations against *S. aureus*, icariin alone did not show any significant antifungal activity *in vitro* (17).

Subsequently, we evaluated the potential synergistic effect of this flavonol in conjunction with representatives from the three most commonly used classes of antifungal compounds, FCZ, amphotericin B and caspofungin, measuring the corresponding MICs of these three compounds against a *C. albicans* laboratory strain. Icariin was found to enhance the activity of FCZ, decreasing the MIC on agar plates from 3 to 1 µg/ml (Figure 1). The potentiation factor relating to the interaction between icariin and FCZ was calculated to be 3, indicating that the relationship is synergistic. This synergistic activity has also been described for other flavonoids such as quercitin, kaempferol, baicalein and glabridin (30). When applied alone, quercitin has a measured MIC value of 128–512 mg/ml, however, this flavonol also shows strong synergism with fluconazole (FCZ) against FCZ-resistant *C. albicans* strains (31). Quercitin was also able to reverse antifungal resistance in FCZ-resistant *Candida tropicalis* isolates through synergy with azoles (30). The flavonol kaempferol (precursor of icariin) exhibited an MIC of 12.5 μg/ml against *C. albicans*, but interestingly, kaempferol was also able to reverse the antifungal resistance observed in FCZ-resistant *C. albicans* strains, when co-incubated with FCZ (30). Quercitin was also able to reverse antifungal resistance in FCZ-resistant *Candida tropicalis* isolates through synergy with azoles (30).

No changes in the MICs of amphotericin B and caspofungin were detected when icariin was incorporated in the agar plates (Figure 1). Co-incubation of *C. albicans* cells in the presence of the three antifungals with and without icariin in YPD agar plates (Figure 2A) and determination of growth in YPD broth (Figure 2B) corroborated our initial findings. Our checkerboard analysis revealed further detail regarding the synergistic relationship between icariin and FCZ (Figures 3A,B). A dose-dependent (in the case of both co-adjuvant and antifungal) inhibition of growth of *C. albicans* was observed. Higher co-adjuvant concentrations resulted in a stronger growth suppression at each antifungal concentration. Interestingly, at 6 h, growth inhibition caused by the highest concentrations of icariin was found to be statistically significant, whilst at the 24 h mark this was true for lower icariin concentrations. A number of studies have been published over the years demonstrating the successful use of natural compounds as co-adjuvants in antifungal therapy, however work has primarily focused on polyphenols and terpenes with the vast majority of the studies being performed *in vitro* (32). Further work is required to elucidate the exact mechanism of action of icariin, although the current data suggest that accumulation of icariin facilitated damage to the cell membrane may play a role in the observed effects.

To further explore the role of icariin as a potential natural adjuvant in antifungal treatments, activity of this flavonol was also evaluated in the presence of ketoconazole and itraconazole, two other components of the azole drug family. Ketoconazole is an imidazole indicated for secondary oral candidiasis, such as chronic mucocutaneous and chronic hyperplastic candidiasis (6). On the other hand, itraconazole is a triazole with activity against *C. albicans*, *C. krusei* and *C. glabrata.* Since these two species are naturally resistant to FCZ, itraconazole emerges as a good alternative with clinical relevance in the treatment of patients infected with fluconazole-resistant *C. albicans* strains. Activity of both azole compounds against *C. albicans* was enhanced when icariin was added to the agar plates, as shown above, confirming the synergistic effect of the flavonol icariin with other members of the azole antifungal family (Figure 4).

According to these findings the proposed mechanisms of action for icariin as an adjuvant in azole regimes is that it functions by facilitating the activity of this antifungal class in damaging cell envelope. This hypothesis was tested in three different conditions—using the two-color fluorescent fungal viability probe, FUN1 in co-culture with stress-inducing agents SDS and calcofluor white which target the cell membrane and the cell wall, respectively. The dye FUN-1 freely permeates fungal plasma membranes into the cell and is distributed in the cytoplasm as a bright diffuse green stain. In active fungal cells, the dye is metabolized into orange/red intravacuolar structures (19). Fluorescence microscopy analysis revealed that we were able to detect red vacuolar fluorescence at an earlier time point in cells grown in the presence of icariin both with and without FCZ, suggesting that icariin may enhance translocation of the dye (Figure 5). This mechanism would in turn enhance the fungistatic action of FCZ. Fluorescence microscopy analysis using propidium iodide reconfirmed previous observations that icariin is non-toxic to the growing fungal cells (Figure 6). There were no significant differences in the proportions of live/dead cells observed between the samples incubated in FCZ vs. FCZ+ icariin. We propose that the clinical importance of icariin lies in the enhancement of the fungistatic effect of FCZ.

To assess whether the suspected increased permeability caused by icariin was located at the cell wall or the plasma membrane serial dilution spot assays were done on YPD-FCZ agar plates containing SDS or CFW. A synergistic effect between icariin and SDS was detected, whereas no effect on growth inhibition of *C. albicans* was detected with the icariin-CFW combination (Figures 7, 8). These two tests allowed us to conclude that it is unlikely that the mechanism of action of icariin involves the cell wall, as growth of *C. albicans* in FCZ-CFW in the presence or absence of the flavonoid was unaffected (Figure 8). We propose that icariin may increase the sensitivity of *C. albicans* to fluconazole via a cell membrane mediated mechanism, as the growth of the yeast was strongly reduced on plates containing SDS + FCZ. These findings correlate with those described by Gazoni et al., where kaempferol has shown inhibition of cell membrane function in the fungi *Neuropospora crassa* (33). One of the antifungal mechanisms exhibit by quercitin includes also disruption of the plasma membrane (34).

As a consequence of the increase in antimicrobial resistance infections in recent years, interest in natural compounds as a source of novel therapeutical agents with antibacterial activities has risen (12–14, 23). Nevertheless, research about new natural antifungal compounds and regimes is not so abundant, even though the prevalence of fungal infections is on the rise (3, 4). Amongst natural compounds, flavonoids have emerged as an interesting class of molecules with promising results to combat oral fungal infections (26, 28, 35). Our findings in regards with the flavonol icariin and its synergistic effect when used in conjunction with azoles represent an interesting avenue in the advancement of antimicrobial stewardship policies, by reducing the amount of antifungal needed in the treatment of oral infections. Icariin is a widely used natural compound in traditional chinese medicine, derived from plants from the genus *Epidemium*, with several interesting pharmacological activities, as well as low toxicity. Future directions of our research include testing the synergistic icariin-FCZ effect on FCZ-resistant oral isolates of *C. albicans*, as well as evaluating *in vivo* the correct dosage of icariin to apply it along the azole regimes.

## Data Availability

The original contributions presented in the study are included in the article/Supplementary Material, further inquiries can be directed to the corresponding author.
